# A cognitive style dataset including functional near-infrared spectroscopy, eye-tracking, psychometric and behavioral measures

**DOI:** 10.1016/j.dib.2019.104544

**Published:** 2019-09-19

**Authors:** R.C.A. Bendall, S. Lambert, A. Galpin, L.P. Marrow, S. Cassidy

**Affiliations:** Directorate of Psychology & Public Health, School of Health Sciences, University of Salford, Salford, UK

**Keywords:** Cognitive style index, Functional near-infrared spectroscopy, Neuroimaging, Eye-tracking, Visual attention

## Abstract

The dataset includes data from the triangulated investigation reported in our paper: ‘Psychophysiological indices of cognitive style: A triangulated study incorporating neuroimaging, eye-tracking, psychometric and behavioral measures’ [1,2]. The data was collected at the Directorate of Psychology & Public Health laboratories at the University of Salford, UK, in 2015 among an English-speaking sample. The dataset includes measures described in the paper including information-processing/cognitive style recorded as Cognitive Style Index [CSI; 3] scores, comparative visual search (CVS) task behavioral measures (reaction time and accuracy), eye-movement data (fixation duration, number of saccades, number of comparative saccades and distance moved) and prefrontal cortex (PFC) oxygenated hemoglobin (oxy-Hb) recorded using functional near-infrared spectroscopy (fNIRS).

**Table:** Specifications Table

Subject area	*Psychology*
More specific subject area	*Cognitive Science, Cognitive Neuroscience, Visual Attention, Individual Differences*
Type of data	*SPSS.sav, SPSS.sps, Excel.xlsx, nir, mrk, edat2*
How data was acquired	*Self-report psychometric questionnaire responses were collected using the Cognitive Style Index*[3]*. Behavioral data was recorded using E-Prime 2.0 (Psychological Software Tools). Eye-tracking data was collected using a Tobii T120 eye-tracker and Tobii Studio software (Tobii Pro). An fNIR Imager 1000 (Biopac Systems Inc.) collected fNIRS data using Cognitive Optical Brain Imaging Studio (fNIR Devices, LLC). fNIRS data was analyzed offline using fnirSoft*[4].
Data format	*Raw, CSI composite scores*
Experimental factors	*The study had a quasi-experimental between-subjects design in which participants were allocated into one of two groups on the basis of their CSI scores; quasi-analytic and quasi-intuitive. Participants then completed the CVS task whilst changes in PFC oxy-Hb and eye-movements were recorded. CVS task stimuli were a subset of real-world scenes selected from a larger stimulus set*[5].
Experimental features	*The dependent variables included behavioral task performance, eye-movement metrics and changes in PFC oxy-Hb.*
Data source location	*University of Salford, Salford, UK.*
Data accessibility	*Data is available with this article.*
Related research article	*Bendall, R. C. A., Lambert, S., Galpin, A., Marrow, L. P. and Cassidy, S. Psychophysiological indices of cognitive style: A triangulated study incorporating neuroimaging, eye-tracking, psychometric and behavioral measures. Personality and Individual Differences, 144, (2019) 68–78.**https://doi.org/10.1016/j.paid.2019.02.034*

**Table:** 

**Value of the Data** • Provides the opportunity for further psychophysiological validation analysis of the CSI, possible in the context of competing dual-process [3] vs. unidimensional bipolar [6], [7] conceptual models of information processing/cognitive style. • The data can be reanalyzed, used for replications studies, and included in future meta-analyses. • Data demonstrates the value of combining psychometric, behavioral, eye-tracking and neuroimaging methods in experimental designs and offers insight for researchers in the development of further experiments.

## 1 Data

The dataset contains psychometric, behavioral, eye-tracking, and functional neuroimaging observations from a quasi-experimental study employing a CVS task [1], [2]. Psychometric data includes self-report scores from the CSI (SPSS.sav). Behavioral data includes response accuracy and response time for the CVS task (E-Data-Aid.edat2). Eye-tracking metrics include fixation duration, number of saccades, number of comparative saccades and distance moved (Excel.xlsx). Neuroimaging data include oxy-Hb recorded with fNIRS (nir, mrk). A combined master data file is also included (SPSS.sav).

## 2 Experimental design, materials and methods

### 2.1 Design

The study adopted a quasi-experimental between-subjects design. The independent variable was information-processing/cognitive style (analytic or intuitive) based upon CSI criterion. Dependent variables included behavioral performance on the CVS task (accuracy and response time), eye-tracking metrics on the CVS task (fixation duration, number of saccades, proportion of comparative saccades and distance moved), and neural activation indexed with changes in levels of oxy-Hb.

### 2.2 Participants

Fifty-six university staff and students (45 female, 11 male) aged 18–57 years (*M* = 28.53, *SD* = 9.48) contributed to the dataset. Informed consent was gained from all participants. Ethical approval was granted from the School of Health Sciences Ethics Panel at the University of Salford (HSRC12-88). All participants were provided with a £10 inconvenience allowance.

### 2.3 Materials

The CSI was administered to assess preferred cognitive (or information-processing) style. 38 statements (17 reverse scored) are presented on a 3-point Likert scale with each item attaining a possible score of 0, 1 or 2. The CSI has a theoretical range of 0 (demonstrating an intuitive style) to 76 (representing an analytic style). The CSI includes thresholds for analytic (53–76), quasi-analytic (46–52), adaptive (39–45), quasi-intuitive (29–38), and intuitive (0–28) styles. The current study created 3 groups by combining participants with scores in the quasi-intuitive and intuitive threshold as one group (0–38), scores in the quasi-analytic and analytic threshold as another group (46–76), and an adaptive group (39–45).

The CVS task was presented with E-Prime and run on a Viglen Intel i7 Core computer with a 120 Hz, 22-inch monitor. 20 randomized trials presented pairs of real-world images in parallel. In 50% of the trials the image pairs were identical and in the remainder a subtle difference existed between the two images. The objective was to identify if a difference was evident between the two images. One alteration was made to the changed images consisting of either a deletion (in part or in full), a change to the location of an object, a change in color, or alterations in orientation. The stimuli are described in detail in [5].

A Tobii T120 eye tracker (Tobii Pro) was used to record eye movements. Data was recorded at a frequency of 120 Hz with a spatial resolution of 0.2°. Tobii Studio (Tobii Pro) software was used to record eye movements and fixations were calculated with the default Tobii-I-VT Fixation Filter [8]. A 9-point calibration was performed for each participant.

Relative changes in PFC oxy-Hb were recorded using fNIRS. A continuous wave 16 channel fNIR Imager 1000 (Biopac Systems Inc.) recorded fluctuations in levels of infrared light at 850nm and 730nm wavelengths using Cognitive Optical Brain Imaging Studio (COBI; fNIR Devices, LLC) at a frequency of 2 Hz. The probe was secured such that the midpoint was aligned with Fpz according to the 10–20 system [9]. Data was analyzed using fnirSoft [4]. Synchronization markers were sent from E-Prime to COBI to allow extraction of baseline and task-related changes in oxy-Hb.

### 2.4 Procedure

Once participants had provided informed consent the fNIRS sensor headband was attached to participants using elastic strapping. Next, baseline brain activation was recorded whilst the participants were resting for 5 seconds. Participants were seated 70cm from the monitor used to display the CVS task and which also housed the Tobii T120 eye-tracker. To minimize head movements allowing for optimal eye-tracking and oxy-Hb data collection all participants used a chin rest. Following successful calibration with the eye-tracker the CVS task began. The task was presented using E-Prime 2.0 on a 22-inch screen with resolution of 1024 × 768 pixels. During the task participants were required to indicate whether the two images presented simultaneously were identical or different. The task was not time limited and participants were asked to respond as promptly as possible once they were confident that their response was correct. There were 4 practice trials and 20 experimental trials. To make their responses participants pressed ‘Q’ if they believed the images were identical and ‘P’ if they believed a difference was present. Participants received feedback at the end of each trial. Finally, at the end of the experiment, participants completed the CSI.

### 2.5 Analysis

For comparative analyses, scores from the CSI were used to create two style groups; quasi-analytic (scores within the range 46–76) and quasi-intuitive (scores within the range 0–38). Participants whose CSI scores identified them as ‘adaptive’ (39–45) were omitted from group-based analyses. However, they were included in correlational analyses.

Response accuracy and response time measures were averaged for each participant across all CVS task trials. One participant was excluded from all analyses due to lack of engagement with the CVS task as evidenced by poor accuracy (35% of trials correctly identified) and a high percentage of false positive responses (80%). Further participants were removed if their accuracy or response time scores exceeded the threshold of 3 standard deviations from the mean resulting in one additional participant from the analytic group being excluded from the response time analyses.

Eye gaze behavior was computed across four parameters for each participant: average fixation duration (1), average number of saccadic eye-movements (2), average proportion of comparative saccades (3), and average distance that gaze moved from corresponding points across images (4). Fixations of less than 100 milliseconds (ms) were first removed before average fixation duration (1) was calculated in ms for each trial, and then an average across trials was calculated for each participant. The number of saccadic eye movements (2) was taken for each trial and again averaged for each participant. An algorithm computed, for each trial, the number of saccadic eye movements that crossed from one image to the other and then divided this by the total number of saccadic eye-movements for that trial. This was averaged across trials for each participant to compute the average proportion of comparative saccades (3). Finally, the distance that each saccade moved from corresponding points (4) was calculated for each trial by subtracting the distance between the two corresponding points on each image (670 pixels) from the horizontal x-coordinate of all fixations on the right-hand image. This allows the second image to be mapped onto the first, providing an indication of how far, in pixels, saccades on the second image were directed away from the corresponding point on the first image. An average was taken per trial before an average across trials was calculated for each participant. Fourteen data sets were lost due to technical failure and a further data set was removed due to poor calibration (substantial periods of unstable signal, and over 40% of fixations less than 100 ms), therefore 41 data sets were available for eye-tracking analysis.

fNIRS raw data were processed as follows. Initially, a finite impulse response linear phase low-pass filter with order 20 and cut-off frequency of 0.1Hz was employed to attenuate high frequency noise, respiration and cardiac effects. Next, visual inspection of the data and a sliding-window motion artifact rejection algorithm were used to remove motion artifacts and saturated channels. Finally, oxy-Hb was then calculated using the modified Beer-Lambert Law [10]. We created two regions of interest representing the right dorsolateral prefrontal cortex comprising channels 11, 12, 13 and 14, and the left dorsolateral prefrontal cortex including channels 3, 4, 5 and 6.

## Funding

This research was supported by funding from the British Psychological Society (Psychobiology Section) and from the University of Salford's Research Impact Fund.
