# Supplementary material for: A cognitive style dataset including functional near-infrared spectroscopy, eye-tracking, psychometric and behavioral measures
Source: Data Brief. 2019 Sep 19;26:104544. doi: 10.1016/j.dib.2019.104544 (PMC6811964; doi:10.1016/j.dib.2019.104544)
Supplement: Multimedia component 1 [file mmc1.docx]

Codebook: A cognitive style dataset including functional near-infrared spectroscopy, eye-tracking, psychometric and behavioral measures

Variable descriptors corresponding to the accompanying master data file (.sav)

| Variable | Description |
| --- | --- |
| Quasi_groups | Participant grouping based upon Cognitive Style Index score; I/QI = quasi-intuitive, A/QA = quasi-analytic, adapt = adaptive |
| CSI_score | Total Cognitive Style Index score |
| Age | Participant age (years) |
| Gender | Participant gender; 1 = male, 2 = female |
| ACC | Average comparative visual search task accuracy score (%) |
| ACC_Group_Filter | Filter for accuracy group analyses; 1 = I/AQ & A/QA (included), 0 = adaptive (excluded) |
| ACC_Correlation_Filter | Filter for accuracy correlation analyses; 1 = included, 2 = excluded. |
| RT | Average comparative visual search task reaction time for all trials (seconds) |
| RT_Correct | Average comparative visual search task reaction time for correct trials (seconds) |
| RTANDRT_Correct_Group_Filter | Filter for reaction time group analyses; 1 = I/AQ & A/QA (included), 0 = adaptive (excluded) |
| RTANDRT_Correct_Correlation_Filter | Filter for reaction time correlation analyses; 1 = I/AQ & A/QA (included), 0 = adaptive (excluded) |
| AverageFixLength | Average fixation length (milliseconds) |
| NumberofSaccs | Average number of saccadic eye-movements |
| DistanceMoved | Average distance moved (degrees) |
| ProportionofSwitches | Average proportion of comparative saccades (%) |
| Eyetracking_Group_Filter | Filter for eye-tracking group analyses; 1 = I/AQ & A/QA (included), 0 = adaptive (excluded) |
| Eyetracking_Correlation_Filter | Filter for eye-tracking correlation analyses; 1 = I/AQ & A/QA (included), 0 = adaptive (excluded) |
| V1 | Average voxel 1 oxygenated haemoglobin change (%) |
| V2 | Average voxel 2 oxygenated haemoglobin change (%) |
| V3 | Average voxel 3 oxygenated haemoglobin change (%) |
| V4 | Average voxel 4 oxygenated haemoglobin change (%) |
| V5 | Average voxel 5 oxygenated haemoglobin change (%) |
| V6 | Average voxel 6 oxygenated haemoglobin change (%) |
| V7 | Average voxel 7 oxygenated haemoglobin change (%) |
| V8 | Average voxel 8 oxygenated haemoglobin change (%) |
| V9 | Average voxel 9 oxygenated haemoglobin change (%) |
| V10 | Average voxel 10 oxygenated haemoglobin change (%) |
| V11 | Average voxel 11 oxygenated haemoglobin change (%) |
| V12 | Average voxel 12 oxygenated haemoglobin change (%) |
| V13 | Average voxel 13 oxygenated haemoglobin change (%) |
| V14 | Average voxel 14 oxygenated haemoglobin change (%) |
| V15 | Average voxel 15 oxygenated haemoglobin change (%) |
| V16 | Average voxel 16 oxygenated haemoglobin change (%) |
| V3_6 | Average voxels 3-6 oxygenated haemoglobin change (%) |
| V11_14 | Average voxels 11-14 oxygenated haemoglobin change (%) |
| Baseline_NIRS_Left | Baseline voxels 3-6 oxygenated haemoglobin |
| Baseline_NIRS_Right | Baseline voxels 11-14 oxygenated haemoglobin |
| NIRS_Group_Filter | Filter for NIRS group analyses; 1 = I/AQ & A/QA (included), 0 = adaptive (excluded) |
| NIRS_Correlation_Filter | Filter for NIRS correlation analyses; 1 = I/AQ & A/QA (included), 0 = adaptive (excluded) |
